# Dental Practice

**Published:** 1840-07

**Authors:** 


### DENTAL PRACTICE;

*Or Observations on the qualifications of the Surgeon Dentist.—Dental Quackery.—Nature, and extent of the duties of Dentists in the first and second dentition.—The defective teeth of the present day compared with those of the last century, owing to empiricism.—Regulation and management of the teeth.—The present absurd and destructive practice of filing teeth.—Treatment of tooth-ache.—Extraction of teeth.—New extracting instruments invented by the author, illustrated by plates.—Third dentition.—Importance of artificial teeth.—Philosophical principles on which they are formed—and on the duty of Surgeon Dentists and Mechanical Dentists, each by themselves to unite in associations, for the purpose of abating dental quackery. By* JOHN GRAY, *Surgeon Dentist, member of the Royal College of Surgeons in London.—London,* 1837.—*pp.* 54.

The work, of which the above is the title, was placed on our table a short time since by a professional friend; and were we to judge of its worth by the length of this title, our opinion of it could not be otherwise than exalted; but unfortunately the perusal of its pages has disposed us to regard it in a very different light. The object of the author in this publication seems to have been any thing rather than the promulgation of new or useful information on either the theory or practice of Dental Surgery.

The introduction, after stating that the author is a member of the Royal College of Surgeons in London,—that it has been his intention for a long time, to present to the profession the result of his own observations and experience, &c., is made up of a recapitulation of the title page. On the qualifications of the dentist and on dental quackery, there are some good observations; from the former, we quote the following:—"It is only when the accomplished mechanic has superadded the qualifications of the surgeon, that he may legitimately assume the whole range of the profession of a dentist with credit to himself and advantage to his patients."

Of the practical parts of the science, the author touches only upon a few, and here his directions are too brief and inexplicit to be of any service either to the general reader or practitioner.

On the instruments used for the extraction he professes to have made some valuable improvements. These consist in a few slight alterations in the old elevator and key instruments, by which, instead of making them any better, they are rendered more clumsy and awkward than they originally were.

The great misfortune with many of the writers on dentistry, in this mercenary age, seems to have been, that they have labored more for the advancement of their own fame, than for that of their art. Many of the works that have been published on this subject, during the last ten or fifteen years, seem rather to be intended as popular advertisements, than as disseminators of new or useful truths in this department of knowledge.

[BALT. ED.

